# The Connection Between Magnesium and Heart Health: Understanding Its Impact on Cardiovascular Wellness

**DOI:** 10.7759/cureus.72302

**Published:** 2024-10-24

**Authors:** Raqshan W Siddiqui, Syed Muhammad Hayyan Nishat, Asma A Alzaabi, Fatema M Alzaabi, Dana J Al Tarawneh, Yusuf J Al Tarawneh, Abdallah Khan, Mohammed Abdul Muqsit Khan, Tabish W Siddiqui, Shiza W Siddiqui

**Affiliations:** 1 Internal Medicine, Ras Al Khaimah (RAK) Medical and Health Sciences University, Ras Al Khaimah, ARE; 2 Research, Dubai Medical College, Dubai, ARE

**Keywords:** cardiovascular disease, coronary artery disease, dietary supplements, hypertension, magnesium

## Abstract

Magnesium is a crucial mineral that supports various enzymatic processes in the body. It plays a vital role in maintaining vascular, metabolic, and electrical homeostasis, making it an important factor for cardiovascular health. Magnesium is also involved in oxidative and inflammatory responses. Low levels of magnesium are associated with several cardiovascular issues, including arrhythmias, coronary artery disease, stroke, high blood pressure, and abnormal lipid levels. This suggests that a deficiency in magnesium could be a risk factor for cardiovascular disease (CVD), which is a major public health concern. Monitoring serum magnesium levels might help in identifying cardiovascular problems and related risk factors. Additionally, magnesium supplementation could lead to new approaches for managing CVDs.

## Introduction and background

With one in four deaths occurring in the United States, cardiovascular diseases (CVDs) have continued to rank among the top two causes of death since 1975 [1]. In 2015, the leading causes of death were cancer and heart disease [1]. According to estimates from the World Health Organization, CVD was the leading cause of death worldwide in 2015, with 17.7 million fatalities [2]. Based on projections, the annual cost of CVD is expected to reach $237 billion by 2035, surpassing even the expenditures of diabetes and Alzheimer's disease, making it the most costly disease overall [2].

The autonomic nervous system, endothelium cells, smooth muscle layer of blood vessel cells, vasa vasorum, and adventitial tissues, including inflammatory cells, are among the various cell components that control vascular function [3]. Vascular function variations, including endothelial dysfunction and arterial stiffness, are significant risk factors for CVD [4]. Present recommendations promote a healthy nutritional routine, which includes Mediterranean-style dietary patterns and dietary approaches to stop hypertension and avoid CVD because there is significant research that shows that food choices are major predictors of risk for CVD [5]. The effects of intake of magnesium on blood vessel function may be partially responsible for the beneficial effects of a nutritious diet on the risk of CVD, albeit the exact processes are still unknown [6].

Magnesium is a plentiful micronutrient and cation, and it activates enzymes, aids in the synthesis of energy, and controls the levels of calcium and associated biomarkers, among other vital functions in the body [7]. Low magnesium levels have been linked to CVD through a variety of physiological processes, including high blood sugar levels, chronic inflammation, hypertension, abnormal tone of the vessels, and circulation to the peripheral tissues [8]. In various settings, total magnesium in the serum has long been used to evaluate magnesium levels [8]. In multiple studies, low levels of magnesium have been linked to a higher risk of various health problems, such as a greater risk of diabetes, hypertension, and CVD [9-11].

The selection of the studies cited in this review was based on their relevance to the clinical implications and pathophysiological mechanisms of magnesium in cardiovascular health. The selection procedure comprised finding research that showed magnesium's impact on a range of CVDs, including lipid profiles, arrhythmias, coronary heart disease, stroke, and hypertension. Important variables were the study designs (e.g., prospective studies and randomized controlled trials), the populations studied, and the particular outcomes assessed in relation to dietary consumption or supplementation with magnesium.

Given the high prevalence of CVDs and their significant public health impact, investigating the link between magnesium deficiency and cardiovascular risk is imperative. Utilizing serum magnesium levels as a cost-effective screening tool may enhance risk stratification in clinical practice. Despite its importance, there remains a gap in systematic research across diverse populations. This review aims to elucidate the relationship between magnesium and cardiovascular health outcomes, ultimately contributing to improved prevention and management strategies for CVD.

## Review

Pathophysiology of magnesium in cardiovascular health

Magnesium improves the function of the cardiovascular system by acting on membrane ion flow pumps, encouraging endothelium-dependent blood vessel dilatation, reducing blood pressure, reducing inflammation, and boosting insulin and glucose breakdown [12]. In addition, magnesium contains antiplatelet and anticoagulant characteristics, is a natural inhibitor of calcium, and is a necessary cofactor in cellular oxidation reactions [12].

Magnesium's Role in Modulating Ionic Channels

Certain ionic channels, including calcium, potassium, and sodium, are controlled in part by magnesium [13,14]. Magnesium regulates cardiac responsiveness and the length of action potentials by decreasing the fast influx element of the delayed rectifier potassium channel [15]. Magnesium influx influences the elongation of QRS and PR duration and also slows atrioventricular node conductivity [16].

Magnesium inhibits coronary vessel spasm, plays a critical function in regulating vascular muscle tone and, consequently, systemic arterial blood pressure, and provides protection against stimulated activity through its antagonistic impact on two calcium channels, transient type (T-type) and long-lasting type (L-type) [17]. Magnesium is also essential for the exchange of potassium for protons as well as for preventing potassium loss [18]. This process is impaired by hypomagnesemia, which also encourages the increase of cytoplasmic sodium and calcium levels [18]. The excess of calcium inside the cells is linked to cardiac ischemia, which negatively impacts cardiac function [19]. In addition to competing for the same binding sites as calcium, magnesium might restrict the extent of the infarct by minimizing coronary vessel contractions, lowering damage from oxidation after myocardial infarction, and enhancing endothelial-dependent dilatation of vessels through the production of nitrous oxide during myocardial ischemia [19,20].

Magnesium’s Role in Enzymatic Reactions

Magnesium transporters are membrane proteins that regulate the influx of magnesium ions into cells in response to physiological signals, ensuring cellular homeostasis [21]. Magnesium also functions as a component in a variety of cellular activities, some of which occur in mitochondria, which also serve as the primary intracellular storage of this ion [21]. Mitochondrial structure and power synthesis are both impacted by disturbances in the equilibrium of mitochondrial magnesium, which results in an impairment in adenosine triphosphate generation [21].

Magnesium’s Role in Metabolic Regulation

By lowering the likelihood of metabolic syndrome and type 2 diabetes mellitus, magnesium supplementation appears to have positive benefits on cardiovascular health [22,23]. The glucose transporter protein 4 is regulated by this ion, which improves insulin responsiveness and increases both insulin and glucose breakdown [24]. Magnesium has been shown to control postreceptor signaling via insulin, mediate insulin release from the pancreas, and function as a second responder for transmitting insulin-mediated signals [25,26].

Magnesium’s Role in Regulating Inflammatory Reactions

Magnesium exhibits antioxidant properties by neutralizing free radicals of oxygen and reduces inflammation by regulating the expression of nuclear factor kappa B [27-33]. In hypomagnesemia, the inflammation that occurs also affects the balance of lipids by lipid peroxidation, which leads to dyslipidemia by raising lipids rich in triglycerides, boosting plasma levels of a protein called apolipoprotein B, and lowering the concentrations of high-density lipoprotein cholesterol (HDL-C) [33,34].

Magnesium’s Role in Hemostasis and Coagulation

**Figure 1:**
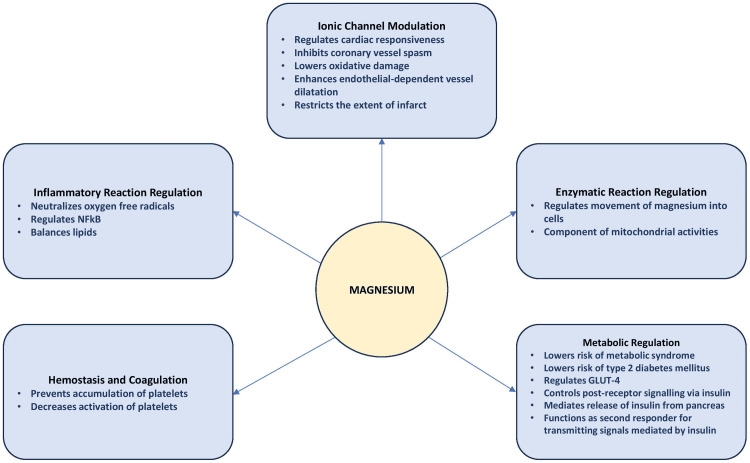
Pathophysiology of magnesium in cardiovascular health NFkB: nuclear factor kappa B; GLUT-4: glucose transporter protein 4 Image credits: The image has been created by the author Raqshan W. Siddiqui

Magnesium can prevent the accumulation of platelets by competing with the ions of calcium for particular places in the glycoprotein (Gp) IIb subunit, changing the receptor's configuration, and preventing interactions between Gp IIb-IIIa and fibrinogen [35]. Additionally, magnesium can decrease the activation of platelets by preventing the synthesis of factors that stimulate platelets, like thromboxane A2, and by promoting the breakdown of factors that inhibit platelets, like prostacyclin (Figure 1) [35,36].

Clinical insights into magnesium's role in cardiovascular health

Association Between Magnesium Supplementation and Cardiac Arrhythmias

A study was conducted by Raghu et al. on 55 participants with a cardiac response rate of more than 120 beats/minute and atrial fibrillation [37]. Of them, 75% received treatment with magnesium sulfate (MgSO_4_) and 25% received a placebo [37]. In addition to conventional therapy, 2.5 grams of intravenous MgSO_4_ was given, and it led to a conversion to sinus rhythm and a reduction in pulse rate [37]. Davey and Teubner observed similar outcomes in a prospective, randomized, double-blind, placebo-controlled experiment on 199 adults with fast atrial fibrillation who were admitted to the emergency room [38]. MgSO_4_ was given to 102 participants, while a placebo was given to 97 [38]. It was shown that the MgSO_4_ group had a higher chance of achieving a heart rate of less than 100 beats/minute and changing to sinus rhythm [38].

Two hundred patients undergoing their first coronary artery bypass grafting procedure were examined by Toraman et al. and randomly assigned to two separate groups [39]. In the group that was administered magnesium, atrial fibrillation after surgery affected two patients (2%), whereas in the control group, it affected 21 patients (21%) [39]. Thus, it was determined that magnesium supplementation during the period before surgery, as well as during the early phase after surgery, significantly lowers the risk of atrial fibrillation following coronary artery bypass grafting [39]. This research was supported by an experimental study done in Iran in which 160 adult individuals undergoing heart surgery with normal hemodynamics and normal sinus rhythm were studied [40]. The results showed a major difference in the occurrence of all arrhythmias between the magnesium-treated and untreated groups [40]. Table 1 presents the relationship between magnesium supplementation and cardiac arrhythmias, as observed in various studies.

**Table 1:** Association between magnesium supplementation and cardiac arrhythmias in different studies IV: intravenous; MgSO_4_: magnesium sulfate; NaCl: sodium chloride

Study	Study design	Population	Participants	Magnesium level supplementation	Type of arrhythmia	Result
Raghu et al. [37]	Prospective, randomized, double-blind, placebo-controlled study	Patients with atrial fibrillation	22	20 mEq (2.5 g, 10 mmol) MgSO_4_ over a 20-minute period, followed by 20 mEq (2.5 g, 10 mmol) IV over 2 hours	Atrial fibrillation	Control of heart rate and conversion to sinus rhythm
Davey et al. [38]	Prospective, randomized, double-blind, placebo-controlled trial	Patients with atrial fibrillation and a ventricular response rate >120 beats/min	199	40 mEq (5 g, 20 mmol) of MgSO_4_ in 100 mL of 5% dextrose solution, with 20 mEq (2.5 g, 10 mmol) given IV over a 20-minute period, followed by the remaining 20 mEq (2.5 g, 10 mmol) IV over the next 2 hours	Atrial fibrillation	Rate reduction and conversion to sinus rhythm
Toraman et al. [39]	Prospective, randomized, controlled trial	Patients who had coronary artery bypass grafting	200	6 mmol MgSO_4_ infusion in 100 mL 0.9% NaCl solution (25 mL/hour)	Atrial fibrillation	Reduced incidence of atrial fibrillation
Naghipour et al. [40]	Prospective, randomized, double-blind, placebo-controlled trial	Healthy adult patients who underwent cardiac surgery	160	30 mg/kg MgSO_4_ in 500 cc of isotonic solution IV over 2 hours	Atrial fibrillation, ventricular fibrillation, and others	Significant decrease in the incidence of all types of postcardiac surgery arrhythmias

Association Between Dietary Magnesium and Coronary Heart Disease and Stroke

Kokubo et al. assessed the dietary magnesium intake of 85,293 Japanese participants aged 45-74 years, free of cancer or CVD, using 138-item food-frequency questionnaires from the Japan Public Health Center-Based Prospective Study [41]. In a study conducted by Abbott et al., a cohort of 7,172 men aged 45-68 participated in the Honolulu Heart Program, where 24-hour food recall techniques were employed to assess magnesium intake [42]. Both studies discovered a link between a lower incidence of coronary heart disease and a higher dietary magnesium consumption [41,42].

Zhang et al. reported results from the Japan Collaborative Cohort Study, which involved 58,615 Japanese adults in the age group 40-79, whose dietary magnesium consumption was measured by food frequency questionnaires [43]. Food magnesium intake was found to be inversely linked with death from coronary heart disease and ischemic strokes [43].

In another study by Larsson et al., 34,670 women in the Swedish mammography cohort, aged 49-83, who answered a questionnaire about their food habits in 1997 were investigated for their consumption of potassium, magnesium, and calcium in relation to their risk of stroke [44]. It was demonstrated that the risk of ischemic stroke was negatively associated with magnesium consumption in women with a diagnosis of hypertension [44]. Table 2 highlights the association between dietary magnesium levels and the risk of coronary heart disease and stroke based on findings from various studies.

**Table 2:** Association between dietary magnesium and coronary heart disease and stroke in different studies CHD: coronary heart disease; JACC Study: Japan Collaborative Cohort Study; JPHC Study: Japan Public Health Center-Based Prospective Study

Study	Study design	Population	Participants	Outcome	Magnesium intake assessment	Result
Kokubo et al. [41]	JPHC Study	Healthy patients aged 45-74 years	85,293	CHD	Self-administered 138-item food-frequency questionnaire	Men's risk of CHD was found to be lower when they consumed higher levels of magnesium through diet
Abbott et al. [42]	Honolulu Heart Program	Healthy men aged 45-68 years	7,172	CHD	24-hour dietary recall methods	An increased magnesium intake through food was linked to a lower risk of CHD
Zhang et al. [43]	JACC Study	Healthy patients aged 40-79 years	58,615	CHD, stroke	Self-administered 33-item food-frequency questionnaire	Higher dietary magnesium consumption was linked to decreased mortality rates from CHD and ischemic strokes
Larsson et al. [44]	Swedish mammography cohort	Women aged 49-83 years	34,670	Stroke	Self-administered 96-item food-frequency questionnaire	Among women with a history of hypertension, magnesium intakes were significantly negatively correlated with the risk of cerebral infarction

Association Between Magnesium Supplementation and Hypertension

In 2014, Rodríguez-Moran and Guerrero-Romero recruited 47 metabolically obese, normal-weight (MONW) participants for a randomized, double-blind, placebo-controlled study [45]. For four months, the treatment group was given 30 mL of magnesium chloride (MgCl_2_) 5% solution, while the untreated group was given 30 mL of placebo solution [45]. A comparable study was conducted by Guerrero-Romero and Rodríguez-Morán in 2009 on persons with diabetes who were hypertensive and had low levels of serum magnesium, were not taking diuretics, and were taking captopril at the same time [46]. At the end of the follow-up, both studies found that the average diastolic and systolic blood pressure changes were considerably lower in the treatment group of patients than in the control group [45,46].

To assess the effects of oral magnesium supplementation, Borrello et al. and Purvis et al. conducted randomized, double-blind, placebo-controlled trials on patients with moderate hypertension and patients with non-insulin-dependent diabetic mellitus (NIDDM), respectively [47,48]. They concluded that people with NIDDM and mild hypertension can lower their systolic blood pressure by taking an oral magnesium supplement [47,48]. Table 3 displays the correlation between several studies' findings on magnesium supplementation and hypertension.

**Table 3:** Association between magnesium supplementation and hypertension in different studies BP: blood pressure; DBP: diastolic blood pressure; MgCl_2_: magnesium chloride; MgO: magnesium oxide; MONW: metabolically obese, normal-weight; NIDDM: non-insulin-dependent diabetes mellitus; SBP: systolic blood pressure

Study	Study design	Population	Participants	Magnesium level supplementation	Result
Rodriguez-Moran and Guerrero-Romero [45]	Randomized double-blind placebo-controlled trial	MONW individuals aged 20-60 with hypomagnesemia	47	30 mL of MgCl_2_ 5% solution for 4 months	Supplementing with oral magnesium lowers BP in MONW patients
Guerrero-Romero and Rodríguez-Morán [46]	Randomized, double-blind, placebo-controlled trial	Diabetic hypertensive adults aged 40-75 with hypomagnesaemia not on diuretic treatment but receiving concurrent captopril	82	2.5 g of MgCl_2_ (50 mL of a solution containing 50 g of MgCl_2_ per 1,000 mL of solution) for 4 months	In diabetic hypertensive adults with hypomagnesemia, oral magnesium supplementation with MgCl_2_ significantly decreases SBP and DBP
Borrello et al. [47]	Double-masked, placebo-controlled, parallel study	Patients with mild hypertension and on a normal salt diet	83	200-mg MgO for 12 weeks	In patients with mild hypertension, magnesium significantly decreases the SBP
Purvis et al. [48]	Randomized, double-blind, placebo-controlled crossover trial	NIDDM controlled by diet and/or an oral hypoglycemic agent, with a serum cholesterol level over 5.20 mmol/L (200 mg/dL); aged 28-84 years	28	384 mg/d MgCl_2_ for 6 weeks	For NIDDM patients, oral magnesium supplementation effectively lowers SBP

Association Between Magnesium Supplementation and Changes in Lipid Profile

According to a 12-week double-blind, placebo-controlled, randomized clinical trial in Iran involving 86 individuals with prediabetes, those who took magnesium supplements had noticeably increased HDL-C levels [49]. Guerrero-Romero et al. observed reduced triglycerides and higher HDL-C in a comparable trial where participants were given 30 mL of a 5% MgCl_2_ or a placebo solution once daily for four months [50].

Solati et al. [51] and Rodriguez-Moran et al. [45] have shown that oral magnesium supplementation enhances the lipid profile in patients with type 2 diabetes and MONW adults, respectively. In the former study, magnesium supplementation was associated with lower levels of low-density lipoprotein cholesterol and non-HDL-C in the patients; in the latter, the magnesium treatment group was associated with lower levels of triglycerides [45,51]. Table 4 illustrates the relationship between magnesium supplementation and alterations in lipid profiles, as observed in various studies.

**Table 4:** Association between magnesium supplementation and changes in lipid profile in different studies BP: blood pressure; GFR: glomerular filtration rate; HDL-C: high-density lipoprotein cholesterol; LDL-C: low-density lipoprotein cholesterol; MgCl_2_: magnesium chloride; MgO: magnesium oxide; MONW: metabolically obese, normal-weight

Study	Study design	Population	Participants	Magnesium supplementation	Outcome	Result
Salehidoost et al. [49]	Randomized double-blind placebo-controlled clinical trial	Prediabetic patients aged 18-65, GFR >60 mL/minute, and BP <140/90 mmHg	86	250-mg MgO tablet once daily for 12 weeks	Increased HDL-C	People with prediabetes had higher HDL-C levels after taking a magnesium supplement
Guerrero-Romero et al. [50]	Randomized double-blind placebo-controlled clinical trial	Prediabetic patients aged 30-65 with hypomagnesemia	116	30 mL of MgCl_2_ 5% solution once daily for 4 months	Decreased triglycerides and increased HDL-C	Supplementing with magnesium helps patients with prediabetes persons' lipid profiles
Solati et al. [51]	Randomized double-blind placebo-controlled clinical trial	Patients with type 2 diabetes aged 20-60 years	54	300-mg elemental Mg daily, for 3 months	Decreased LDL-C and decreased non-HDL-C	For patients with type 2 diabetes, oral magnesium supplementation improves lipid profiles
Rodriguez-Moran et al. [45]	Randomized double-blind placebo-controlled trial	MONW individuals aged 20-60 with hypomagnesemia	47	30 mL of MgCl_2_ 5% solution for 4 months	Decreased triglycerides	Supplementing with oral magnesium improves MONW persons' lipid profile

Limitations

The previously mentioned studies on magnesium and cardiovascular health are subject to a number of limitations that may impact the generalizability and consistency of the results. These limitations include variations in study designs, magnesium dosages, and measurement techniques. In addition, it is difficult to reach firm conclusions because of the wide range of groups examined and the variable lengths of interventions. Numerous studies also have drawbacks, like inadequate confounding variable control, bias risk, and an emphasis on immediate results. Future studies should standardize procedures, incorporate a range of demographics, and investigate the long-term impacts and underlying processes of magnesium's influence on cardiovascular health to overcome these problems.

Future applications

Prospective investigations into magnesium and cardiovascular well-being could yield numerous useful outcomes. Based on unique cardiovascular risk factors, genetic predispositions, and health profiles, customized supplement plans could be created. With this strategy, magnesium supplementation would be customized to optimize benefits and reduce potential side effects for a range of patient types. Moreover, dietary recommendations for magnesium intake might be included in integrated dietary guidelines, which would aid in the management and prevention of cardiovascular disorders. Through dietary plans and educational efforts, public health initiatives might possibly use these findings to raise awareness among at-risk populations and boost their intake of magnesium. This study may lead to improved diagnostic tools that will enable earlier identification of cardiovascular problems associated with magnesium imbalance or insufficiency. Finally, to determine the long-term effects of magnesium supplementation on cardiovascular health, as well as the safety and advantages of doing so over time, long-term clinical research is necessary.

## Conclusions

Because it affects vital physiological processes such as ionic channel modulation, enzymatic reactions, metabolic regulation, inflammation, and hemostasis, magnesium is essential for cardiovascular health. The benefits of it are currently supported by evidence, but more research is necessary because of the limitations of the studies that have already been done, including differences in dosage, study designs, and measurement techniques. Conducting long-term studies, incorporating varied populations, and standardizing research methodologies will yield more conclusive information about magnesium's role in cardiovascular health. A greater understanding could result in more effective public health initiatives, individualized treatment plans, and dietary recommendations, all of which would improve the prevention and management of CVD.
